# Bias, Randomization, and Ovarian Proteomic Data: A Reply to “Producers and Consumers”

**Published:** 2007-02-26

**Authors:** Keith A. Baggerly, Kevin R. Coombes, Jeffrey S. Morris

**Affiliations:** Department of Biostatistics, U.T. M.D. Anderson Cancer Center, Houston, Texas, USA

**Keywords:** calibration, experimental design, mass spectrometry, proteomics, serum profiling

## Abstract

Proteomic patterns derived from mass spectrometry have recently been put forth as potential biomarkers for the early diagnosis of cancer. This approach has generated much excitement, particularly as initial results reported on SELDI profiling of serum suggested that near perfect sensitivity and specificity could be achieved in diagnosing ovarian cancer. However, more recent reports have suggested that much of the observed structure could be due to the presence of experimental bias. A rebuttal to the findings of bias, subtitled “Producers and Consumers”, lists several objections. In this paper, we attempt to address these objections. While we continue to find evidence of experimental bias, we emphasize that the problems found are associated with experimental design and processing, and can be avoided in future studies.

## Background

Proteomic patterns derived from mass spectrometry have recently been put forth as potential biomarkers for the early diagnosis of cancer. Most of the attention has focused on the variant of mass spectrometry known as SELDI-TOF (surface-enhanced laser desorption and ionization time-of-flight) applied to samples derived from easily available biological fluids such as serum or urine. This approach has generated much excitement, particularly in light of results initially reported in The Lancet (Petricoin et al. 2002), suggesting that near perfect sensitivity and specificity could be achieved in diagnosing ovarian cancer using serum samples. In addition to publishing these initial results, the NCI/FDA Clinical Proteomics Program has also made the raw spectra they used available on their web site: http://home.ccr.cancer.gov/ncifdaproteomics/ppatterns.asp. The data from the initial study were soon followed by data from two further SELDI serum studies on ovarian cancer, and most recently, by more high-resolution data derived from a different type of mass spectrometry (Qstar-TOF). In all cases, the posted results match or exceed those from the initial study. These latter datasets have now served as the basis for further papers showing various ways in which ever better separation between cancers and controls can be achieved (e.g., Alexe et al. 2004, Zhu et al. 2003, Conrads et al. 2003,^2004^).

Recently, however, two groups (Sorace and Zhan 2003, Baggerly et al. 2004a) have independently noted that much of the structure present may be due to experimental artifacts that could be introduced, for example, by imperfect randomization of the order in which the samples were run. If this interpretation is correct, then structure associated with bias could confound any meaningful biological information contained in the spectra. In the presence of confounding, said ovarian spectra cannot be accepted as proof that proteomic profiling can reliably be used for cancer identification.

In response, the NCI/FDA group has issued a rebuttal (Petricoin et al. 2004) listing several objections to the findings of bias. The rebuttal notes that these findings “highlight the dangerous potential for error propagation that may arise if a disconnect is allowed to exist between the data producers and the data consumers”. The authors suggest that in order to “prevent the dissemination of inaccuracies and speculative conclusions, we believe that the producers of genomic and proteomic data should be intercalated more fully into the publication process, particularly when the focus of the publication is the analysis of data that the submitting authors have not generated”. This rebuttal has appeared in print as a commentary to the article of Sorace and Zhan (2003), and we refer to it in this article as “Producers and Consumers”.

Our goal is to address the points made in “Producers and Consumers”, specifically those that relate to issues raised in Baggerly et al. (2004a). We do not dispute that one can mathematically analyze these spectra and find algorithms which differentiate cancer and control spectra. Rather, we contend that differences between cancer and control spectra can arise from factors that are not biologically relevant if great care is not taken with the design of the study.

To clarify the notation, we note that there are three SELDI ovarian data sets under discussion:

- DS1: The initial data from the Lancet article,
- DS2: A second set of spectra derived from the same biological samples, but run on a different chip type, and
- DS3: A third set of spectra derived from new biological samples but run on the same chip type as DS2.

All of the data are available from http://home.ccr.cancer.gov/ncifdaproteomics/ppatterns.asp.

## Objections and Responses

We will now try to address the specific objections identified in “Producers and Consumers”. The objections presented in “Producers and Consumers” that relate to points made by Baggerly et al. (2004a) are itemized below. After each, we respond with emphasis on our main contention: that the structure in these data are just as likely to reflect experimental bias as they are to reflect meaningful biological patterns of protein expression.

### 1. Findings in the low M/Z range are dangerous

The first group of objections relates to discussions of findings in the low m/z range of the proteomic spectra, and thus primarily concern DS3.

Both Sorace and Zhan (2003) and Baggerly et al. (2004a) noted that it was possible to perfectly separate cancer spectra from control spectra in DS3 using the intensities at just two m/z values: 2.79 and 245.2. Both of these values are in regions of the spectra that can be very unstable in a medium mass-range (m/z 0–20000) SELDI scan, so the strength of the separation was taken as prima facie evidence of non-random processing (bias).

However, as noted in “Producers and Consumers”:

- it can be dangerous to read much into structure found at very low m/z values in these scans, as such m/z values are outside the range of the calibrants used;
- if the cancer and control samples were randomized, then systematic biases associated with machine jitter should be precluded;
- there may be structure in the low mass proteome which could generate separating structure;
- extension of the presence of bias at 2.79 to the rest of the dataset or to other datasets is “judgmentally biased”.

In general, we agree that using trusting intensities at m/z values outside of the calibration range is a bad idea if one is seeking accurate classification. The m/z values will not be well measured, making later identification of the peptides involved harder, and we may be looking at regions affected by matrix noise (small particles not associated with the samples themselves) if we get to very low m/z values (as we do here). We note, however, that such values were used for classification in both the initial Lancet publication and with the initial postings of the raw data.

Nonetheless, we think that attempts to classify spectra using readings “outside the range” are valuable as negative tests, in part so that we can see how much better our predictions are when we think that some structure should be present than when we think none exists. This is the sense in which we made use of these low m/z intensities.

With respect to randomization in DS3, “Producers and Consumers” notes that “if the investigators would have contacted us, we could have elaborated, as previously stated on our website, that the SELDI-TOF MS data was produced by randomly commingling cases and controls. On any given 8 spot ProteinChip array, both cases and controls were applied in random spot locations”. Thus, they maintain that the values at 2.79 cannot be due to bias.

In general, we agree that proper randomization of the type described should preclude biases associated with a nonrandom sample distribution. However, whatever was previously stated, the website now (Dec 2004) states that the samples “were *not* randomized so that we could evaluate the effect of robotic automation” (emphasis ours). Further, we note that an identical comment about randomly commingling cases and controls was made in the Lancet paper with regard to DS1. One of the findings of Baggerly et al. (2004a) was that a subset of these samples had clearly not been randomized. This finding was not addressed in “Producers and Consumers”.

Having discounted bias, “Producers and Consumers” concludes that the observed structure must be due to real biology associated with the low-mass proteome, which is currently not well understood. While we concede that the low mass proteome has yet to be fully explored, we note that this explanation still seems odd with respect to the peak at 2.79. The signal is very weak, and there are no other peaks nearby in the spectrum. Even a metabolite should have a mass on the order of a single amino acid, and in this mass range there should be other artifacts present.

However, the assumption that the low m/z findings must be biologically relevant rests on the assumption that the data were randomized (addressed above). Rejection of the prior assumption of randomization means that the differences may be due to biology, or they may be due to artifacts; the situation is indeterminate.

Then there is the issue of our judgement. In discussing the structure found in DS3, “Producers and Consumers” notes that Sorace and Zhan’s (2003) interpretation of bias at m/z 2.79 is extended to “the entire SELDI-TOF MS data set, including many other datasets that they did not in fact analyze”, and that these “broad conclusions are judgmentally biased and scientifically unfounded”.

We fail to see how extending the presumption of bias to the rest of the data set is judgmentally biased or even avoidable. Certainly with DS3, if structure at 2.79 shows that the samples were processed differently in some way, that difference should be expected to persist for all m/z values.

As to the latter part of the assertion regarding the other datasets, all three datasets are surveyed in Baggerly et al. (2004a), and the assertions of bias there are based on the analysis of all three.

As our calculations are publicly available, we invite the scientific community to reproduce them in sufficient detail to be satisfied that they are not “scientifically unfounded”.

There is a final semantic issue of whether we are confusing “noise” with “bias” in our investigations of the low m/z region. As we see it, areas where only “noise” (complete lack of structure) is expected can, if they show such unexpected structure, suggest “bias”, e.g. in the form of nonrandom sample allocation to spots or differential preprocessing.

The producers also raise other objections that encompass DS1 and DS2 as well.

### 2. The SOP the producers follow with respect to calibration means that the data are correctly calibrated

“Producers and Consumers” notes that while Baggerly et al. (2004a) “wondered .. about our calibration method, we adhere to strict SOPs whereby any TOF MS is calibrated at the beginning of every analysis”.

We do not dispute that a strict SOP was followed for calibration. However, we believe that the posted values are wrong. The posted spectra show m/z values corresponding to the default calibration that ships with the SELDI software. To us, this mistake suggests an error in file export rather than a failure to attempt calibration, but an error, nonetheless. We have encountered this type of problem ourselves, when we meant to “apply” a calibration equation to all spectra in a set. We accidentally clicked a bit early, and the calibration was applied only to the one clicked spectrum. Consequently, we check both for consistency and for numbers associated with the default settings.

**Figure 1:**
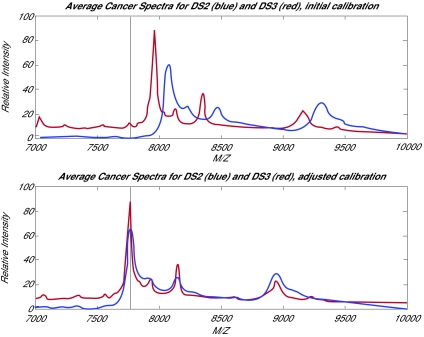
(a) The average cancer spectra from DS2 and DS3, with the location of the maximum peak from Conrads et al. (2003) shown. The posted spectra appear offset. (b) The corresponding average spectra after using the labeled peaks in the Conrads et al. (2003) figure to recalibrate the spectra. Agreement between DS2 and DS3 is now good throughout the region bracketed by calibrants.

As further evidence that a calibration problem exists, we note that in Conrads et al. (2003), where the NCI/FDA Qstar spectra were first described, Figure 4 of that paper shows Qstar and SELDI spectra derived from the same SELDI chip. The chips used for the Qstar experiment were of the same type as those used in DS2 and DS3. In the Conrads et al. (2003) figure, the maxima of the SELDI and Qstar spectra are roughly aligned, and we believe that the alignment shown there is correct. However, if we superimpose the location of the biggest SELDI peak from the Conrads et al. (2003) picture on a plot of the average cancer spectra from DS2 and DS3, we note that the posted maxima are hundreds of units away. This is shown in Figure 1a of this response. If we use the marked peaks in the Conrads et al. (2003) SELDI figure to supply an external calibration for DS2 and DS3, the peak locations are aligned even at m/z values not used in the calibration, as shown in Figure 1b.

One more indicator can be derived from the DS3 spectra. The overall maximum peak is located at m/z 7966 in the average cancer spectrum. Due to the occurrence of multiple charge states (the peptide capturing 2 protons instead of 1), we would expect to see a corresponding peak near m/z 3983. This peak is visible, but at m/z 3993.

The effect of using the default calibration is not slight. The m/z values for DS3 are off by about 2.5% in the vicinity of the biggest peak, and the m/z values for DS2 are off by about 3.9%. As the SELDI results are nominally accurate to within a few tenths of a percent, miscalibration this severe can actively mislead investigators performing database searches based on the reported m/z values.

### 3. One group can find transcendent structure, and another cannot

“Producers and Consumers” notes that while Baggerly et al. (2004a) noted “the inability of features to transcend separate data sets”, a second article by Zhu et al. (2003) “concluded that transcendent features could be found”. The producers cite the latter publication as evidence that DS2 and DS3 contain reproducible biological structure.

Baggerly et al. (2004a) assumed that the errors in calibration described above should preclude the persistence of biological structure across datasets. We verified that the patterns supplied on the NCI/FDA web site did not represent reproducible structure across DS2 and DS3. But, given the offset, we did not conduct an exhaustive search. On the other hand, Zhu et al. (2003) noted that when the 18 m/z values that were chosen to separate cancers from controls in DS2 were used in DS3, perfect separation was observed even though DS3 had been treated as a blinded test set.

This apparent contrast can in fact be easily resolved. The exact approach is detailed in Baggerly et al. (2005), but the key point is simply that DS3 is so easy to correctly classify that near-perfect separation results are obtained using 18 m/z values chosen completely at random.

Thus, biology is not required to explain the separation observed. Further, when the patterns of protein expression at the 18 m/z values supplied are checked in both DS2 and DS3, the directionality of expression changes for 13 of the 18: if expression is higher in cancers in DS2, it is higher in controls in DS3. This suggests that a biological explanation is not only unnecessary to explain the findings in Zhu et al. (2003), it is actively precluded.

### 4. The focus of the objections has been the SELDI data, not the Qstar data

The focus of the analysis in both Sorace and Zhan (2003) and Baggerly et al. (2004a) was on the ovarian SELDI data. However, the producers feel that the more recent high-resolution Qstar data is the current state of the art, and they suggest that more attention should be paid to the better data.

This observed focus is not due to a lack of interest in the Qstar data, but rather to the time lag associated with publication. However, as the Qstar ovarian spectra are derived from SELDI chips, biases that affect these chips can affect the Qstar data as well, so understanding how experimental design issues can affect the SELDI results is still relevant. We note that the file names of the DS3 SELDI spectra are identical to the file names of the Qstar spectra, which suggests to us that the DS3 chips were used in the Qstar experiment. If this is in fact the case, biases affecting the DS3 chips are even more directly relevant.

Further, while the Qstar data are of higher resolution, they also show signs of experimental bias.

**Figure 2:**
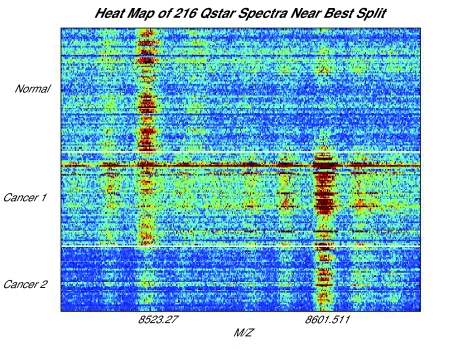
A heat map of the Qstar spectra we have available, sorted by file name, in the vicinity of m/z 8602. This m/z value is identified on the NCI/FDA website as useful for separating healthy women from ovarian cancer patients, and this separation is visible. However, roughly 80 Da below, there is a peak that serves to separate the healthy women and the first half of the ovarian cancer patients from the second half of the ovarian cancer patients.

In Figure 2, we show a heat map of all of the Qstar spectra we have available, sorted by the file names supplied, in the vicinity of m/z 8602. This value is identified in Conrads et al. (2004) as being of use for distinguishing ovarian cancer patients from healthy controls, and a higher level of expression is observed for the cancer patient spectra. However, there is also a visible peak roughly 80 units lower in which expression is high for healthy women but for just half of the cancer patients. As noted in Baggerly et al. (2004b), there is a simple explanation: all of the controls were run before all of the cancers, and a machine breakdown preferentially affected spectra run later in the process.

In response, Liotta et al. (2004) state that “the experimental design element that they highlight in their criticism *was explicitly planned into the study* we reported … We have never claimed or intimated that the samples were randomized and/or co-mingled in the initial experimental design”. (Emphasis ours.)

In our view, however, claims of 100% sensitivity and specificity (as made in Conrads et al. 2004) have meaning only if known sources of variation such as run order have been balanced or randomized. Such claims can be actively misleading if one has chosen to completely confound an effect of interest (cancers vs controls) with run order rather than to randomize.

## Concluding Remarks

The producers have claimed that the consumers are mistaken as to the presence of bias. We respectfully disagree. We are willing to revise our beliefs when features in the data that refute our claims are presented. Until that time, we must repeat our initial position: No one disputes that structure can be found in all of these datasets. However, the structure appears to be associated with strong evidence of experimental bias. As such, the demonstration of structure does not constitute proof that these spectra can be used for clinically meaningful tasks such as the diagnosis of cancer.

We emphasize, however, that the problems described herein are associated with experimental design and analysis techniques, and not with the proteomic technology. With careful design, bias and confounding can be avoided.

In the context of design, we feel that the problems noted to date strongly suggest the need for standards on incorporating information such as run order and clinical information into the reporting of proteomic data. The Microarray Gene Expression Data Society (MGED) has developed such a standard for micro-array data: the Minimum Information About a Microarray Experiment (MIAME; Brazma et al. 2001, Spellman et al. 2002). Exactly what should be supplied in the proteomic equivalent is, we believe, a productive area for debate. Indeed, this was also the consensus of the participants at the Early Detection Research Network (EDRN) meeting on the analysis of SELDI/MALDI data (Seattle, 2004). In the interim, we note with respect to SELDI that current versions of the Ciphergen software support exporting the data in an XML format that could serve as a template for an eventual standard.
